# The Role of Frailty and Myosteatosis in Predicting All-Cause Mortality in Older Adults with Cancer

**DOI:** 10.3390/curroncol31120578

**Published:** 2024-12-06

**Authors:** Efthymios Papadopoulos, Andy Kin On Wong, Sharon Hiu Ching Law, Sarah Costa, Angela M. Cheung, Dmitry Rozenberg, Shabbir M. H. Alibhai

**Affiliations:** 1 School of Kinesiology, Louisiana State University, Baton Rouge, LA 70803, USA; epap@lsu.edu; 2 Centre of Excellence in Skeletal Health Assessment, Joint Department of Medical Imaging, University Health Network, Toronto, ON M5G 2C4, Canada; andy.wong@uhn.ca (A.K.O.W.); 20shl1@michener.ca (S.H.C.L.); sarah.costa@uhn.ca (S.C.); angela.cheung@uhn.ca (A.M.C.); 3 Division of Epidemiology, Dalla Lana School of Public Health, University of Toronto, Toronto, ON M5T 3M7, Canada; 4 Department of Medicine, University Health Network, Toronto, ON M5G 2C4, Canada; 5 Toronto General Hospital Research Institute, University Health Network, Toronto, ON M5G 2C4, Canada; dmitry.rozenberg@uhn.ca; 6 Ajmera Transplant Center, University Health Network, Toronto, ON M5G 2C4, Canada; 7 Division of Respirology, Department of Medicine, Temerty Faculty of Medicine, University of Toronto, Toronto, ON M5S 3H2, Canada; 8 Department of Supportive Care, Princess Margaret Cancer Centre, Toronto, ON M5G 2C4, Canada

**Keywords:** frailty, myosteatosis, muscle density, survival, cancer, older adults

## Abstract

Frailty and myosteatosis are each prognostic of all-cause mortality (ACM) in patients with cancer. However, it is unclear whether myosteatosis adds value to frailty for predicting ACM. We assessed whether myosteatosis improves the predictive ability of frailty for ACM in older adults undergoing chemotherapy. This was a retrospective study of older adults (≥65 years) initiating chemotherapy between June 2015 and June 2022. Frailty was assessed using a 24-item frailty index (FI). Myosteatosis was evaluated via computed tomography scans at the third lumbar vertebra (L3).. Multivariable Cox regression and Uno’s c-statistic determined the predictive performance of the FI and myosteatosis. In total, 115 participants (mean age: 77.1 years) were included. Frailty alone (adjusted hazards ratio (aHR) = 1.68, 95% confidence intervals (CIs) = 1.03–2.72, *p* = 0.037) and myosteatosis alone (aHR = 2.14, 95%CI = 1.07–4.30, *p* = 0.032) exhibited similar performance (c-statistic = 0.66) in predicting ACM in multivariable analyses adjusted for age, sex, body mass index, and treatment intent. However, the highest predictive performance for ACM was observed after inclusion of both myosteatosis and frailty in the multivariable model (c-statistic = 0.70). Myosteatosis improves the performance of frailty for predicting ACM in older adults with cancer. Prospective studies to assess the effect of exercise on myosteatosis in older patients are warranted.

## 1. Introduction

Frailty is a multifactorial geriatric syndrome that affects approximately 42% (range: 6–86%) of older adults with cancer [1]. Frail older patients are more vulnerable to adverse outcomes, such as chemotherapy toxicity, treatment intolerance, postoperative complications, and declines in physical function, in addition to all-cause mortality compared to their non-frail counterparts [1,2]. A precise definition of frailty remains challenging [3], but traditionally, frailty is defined as a multifactorial syndrome characterized by diminished strength, endurance, and reduced physiological function that increases an individual’s vulnerability for developing increased dependency and/or death [4]. A different definition by Rockwood et al. considers frailty as the imbalance between an individual’s assets and ageing-associated deficits [5]. The assessment of frailty per this definition relies on the development of a frailty index (FI) where the number of health deficits present is divided by the total number of health deficits assessed to classify individuals as frail or non-frail using specific cut-offs [6,7,8]. The FI has been shown to predict all-cause mortality in older adults with cancer [7], and although it is considered one of the gold standard methods for assessing frailty, its implementation in daily clinical practice is rare, potentially due to time constrains [9].

Frailty often coincides with impairments in the architecture and composition of the skeletal muscle, such as myosteatosis which describes the infiltration of fat in the skeletal muscle. Myosteatosis is highly prevalent in patients with cancer [mean: 48%, range: 11–85%] and is gaining attention as a prognosticator of shorter survival in individuals with solid and hematological malignancies [10]. Specifically, a meta-analysis of 21,222 adult patients with mixed cancers found a 75% greater risk of all-cause mortality for those with myosteatosis [10]. Myosteatosis is often assessed through computed tomography (CT)-derived skeletal muscle density (SMD) in Hounsfield units (HUs), with lower HU values signifying a higher degree of myosteatosis due to the presence of fat [10,11].

Both frailty and myosteatosis share physical and physiological impairments that may synergistically increase the risk of adverse outcomes including all-cause mortality. For example, muscle strength and low gait speed appear to be common characteristics of both frailty and myosteatosis in patients with and without cancer [12,13,14]. Additionally, insulin resistance and inflammation are each associated with frailty [15,16], myosteatosis [11,17,18], and all-cause mortality [9,19,20].

Emerging evidence in oncology suggests that frail patients with cancer have a higher degree of myosteatosis compared to their non-frail counterparts [12,21]. Specifically, a cross-sectional study of 184 patients with colorectal cancer (mean age: 60 years) found a higher prevalence of frailty in the group of patients with the highest degree of myosteatosis [12]. Similarly, a cross-sectional study of 162 older adults with mixed cancers demonstrated that for each 5-HU decrease in SMD (i.e., higher degree of myosteatosis) there was a 14–20% higher risk of frailty [21]. Whether CT-based myosteatosis can add value to frailty measured by an FI for predicting overall mortality in older adults with cancer is currently unknown. The primary objective of this study was to examine the prognostic value of each parameter, the FI and myosteatosis on all-cause mortality in older adults with cancer initiating chemotherapy. The secondary objective was to assess whether myosteatosis adds value to the FI for predicting all-cause mortality.

## 2. Materials and Methods

### 2.1. Study Setting and Population

We conducted a retrospective cohort study of older adults (≥65 years old) who had undergone chemotherapy at the Princess Margaret Cancer Centre, Toronto, Canada. Data were pooled from two cohorts of patients. The first cohort comprised older adults who were seen in the Older Adults with Cancer Clinic (OACC) for a geriatric assessment prior to chemotherapy between June 2015 and June 2022. The second cohort included older men who had undergone chemotherapy for metastatic castrate-resistant prostate cancer (mCRPC) at the same cancer centre between July 2015 and April 2019 [22].

Participants were included in the analysis if they were ≥65 years of age, had a confirmed diagnosis of either gastrointestinal, genitourinary, or gynecological cancer or lymphoma, had an available abdominal CT scan prior to chemotherapy, had been treated with chemotherapy, had available body height and weight information prior to chemotherapy, and had no missing data on measures of muscle strength and physical performance prior to treatment. In the cohort that underwent geriatric assessment, muscle strength and physical performance were assessed prior to treatment by a clinical nurse specialist using the grip strength test (Jamar grip strength dynamometer) and the Short Physical Performance Battery (SPPB), respectively [23]. Similarly, the grip strength test was used to assess muscle strength prior to chemotherapy in the cohort of older patients with mCRPC. However, physical performance in patients with mCRPC was evaluated based on the 4 m gait speed [22]. Assessment of muscle strength and physical performance for patients with mCRPC was conducted by a trained research coordinator using standard procedures [24].

### 2.2. Data Collection

Data of older adults who had a geriatric assessment in the OACC and received chemotherapy between June 2015 and June 2022 were retrieved from the OACC database combined with electronic medical records. The OACC database contains information on patients’ disease characteristics and domains of the geriatric assessment to inform downstream management strategies and improve patient care [25]. Data of older patients who were treated with chemotherapy in the mCRPC cohort were retrieved from prior studies [22,26]. Prior to chemotherapy, older patients with mCRPC underwent a baseline assessment that evaluated their muscle strength, physical performance, and instrumental activities of daily living (IADLs), as well as the presence of comorbidities. Electronic medical records of patients with mCRPC were used to obtain blood marker values (albumin, alkaline phosphatase, hemoglobin, lactate dehydrogenase, neutrophil and lymphocyte count) and an abdominal CT scan that was performed prior to chemotherapy. All study retrospective data extraction and analyses were approved by the Research Ethics Board at the University Health Network (ID: 22-5600), while the requirement for obtaining informed consent from study participants was waived.

### 2.3. Overall Mortality

Time to all-cause mortality in months was assessed from chemotherapy initiation until June 2022 for older adults in the OACC database and May 2021 for patients with mCRPC. Survival status was confirmed by the Princess Margaret Cancer Registry in addition to obituary searches.

### 2.4. Assessment of Frailty and Myosteatosis

An FI was developed to evaluate the presence of frailty in study participants in line with Searle et al. [6]. The FI comprised 24 variables, including comorbidities, body mass index (BMI), IADLs, cognitive impairment, grip strength, physical performance, and routine blood markers. The variables along with the thresholds used to determine the presence of a deficit are listed in Table S1. The number of deficits present was divided by the total number of deficits assessed to classify participants as non-frail (FI score: ≤0.25) or frail (>0.25) per previous research [8].

Myosteatosis was assessed through an abdominal CT scan at the mid-point of the third lumbar vertebra (L3). SMD was used to quantify the degree of myosteatosis in study participants using two consecutive transaxial CT slices (0.82 × 0.82 × 2.5 mm voxel size). A lower SMD value is thought to represent greater fat infiltration into muscles since fat has a lower attenuation coefficient than muscle. A previous study by Wong et al. demonstrated that up to 49.3% variance in SMD can be explained by muscle adiposity differences, as validated against inter- and intra-muscular fat direct measurement on T1-weighted MRI [27]. The segmentation of skeletal muscle was conducted through an iterative-threshold-seeking algorithm (ITSA) [28] using Jupyter Notebooks (Python 3.9). This algorithm automatically and consistently selects an unbiased threshold to separate muscle from surrounding fat and bone tissues by applying an initial seed threshold using the Otsu algorithm, followed by iterations of noise removal and threshold refinement until the new value is no longer different from the previous. The benefit of using this approach is that it makes no assumptions about the expected density of muscle across the cohorts, which may differ according to patient physiology or scanner cross-calibration. For cases requiring manual correction of muscle boundaries, a region-growing algorithm guided by the previously accepted HU range for skeletal muscle of −29 to +150 [29] was used on SliceOmatic software (Version 6 Rev-9c3, Tomovision, Montreal, QC, Canada) [22]. Myosteatosis was defined using the criteria of Martin et al. [30]. Specifically, participants were classified as having myosteatosis based on the following criteria: (i) SMD < 41 HUs for males and females with a BMI between <20 and 24.9; (ii) SMD < 33 HUs for males and females with a BMI ≥ 25 [30].

### 2.5. Statistical Analysis

Data from the two cohorts were combined in the analysis to increase the sample size. Continuous data were summarized using the mean and standard deviation or the median and interquartile range as appropriate, whereas frequencies and proportions were used to describe categorical variables of study participants at baseline. Differences between frail and non-frail participants prior to chemotherapy were assessed using independent sample t-tests and chi-square tests for continuous and categorical data, respectively. Kaplan–Meier plots and log-rank tests were used to assess the probability of all-cause mortality by frailty and myosteatosis status separately. Multivariable Cox regression models evaluated the associations between the predictor variables (FI and myosteatosis) and the time to all-cause mortality separately. The proportional hazards assumption was assessed visually by ensuring no mild-curve crossing in Kaplan–Meier plots. All multivariable models were adjusted for age, sex, and BMI in addition to other explored variables that were marginally significant correlates of all-cause mortality (*p* < 0.10) in univariable analyses. Specifically, in the first multivariable model, the FI was used as the predictor, adjusting for age per 10-year increase (continuous), sex, BMI, and palliative treatment intent as covariates. In the second multivariable model, myosteatosis was the primary predictor while adjusting for the same covariates. Uno’s c-statistic was used to determine the classification accuracy of FI and myosteatosis in univariable and multivariable models. Lastly, the third multivariable included both the FI and myosteatosis plus the same covariates above to further examine their ability to independently predict all-cause mortality. The interaction between the FI and myosteatosis was also examined in multivariable analysis. All analyses were performed using IBM SPSS for Windows, Version 29.0. Armonk, NY, USA: IBM Corp. Uno’s c-statistic was computed separately in SAS 9.4 (Cary, NC, USA).

## 3. Results

Of the 115 eligible participants (mean age 77.1 years, 71.3% male), 59 (51.3%) were classified as frail and 56 (48.7%) were classified as non-frail (Table 1). Frail and non-frail participants were not significantly different in terms of age, disease site and stage, treatment intent, and type of chemotherapy agent used (Table 1). However, frail participants had significantly higher BMI, specific comorbidities, and a worse blood profile compared to their non-frail counterparts (Table 1). Additionally, of the 59 frail participants, 48 (81.4%) presented with myosteatosis. Similarly, myosteatosis was present in 44 (78.6%) non-frail participants (Table 1). Most frail (66.1%) and non-frail (60.7%) participants had metastatic disease and the majority of this cohort had been diagnosed with genitourinary cancer (Table 1).

### Risk of All-Cause Mortality

**Figure 1:**
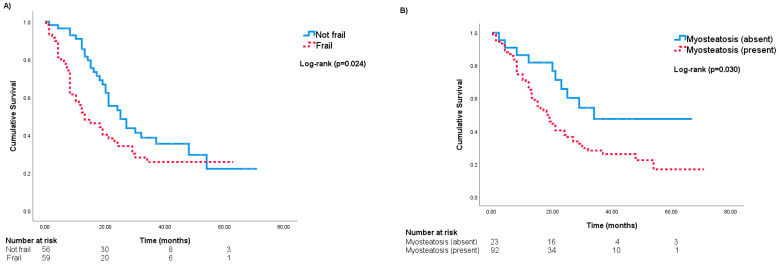
Probability of all-cause mortality based on (**A**) frailty and (**B**) myosteatosis.

Figure 1A depicts the probability of all-cause mortality among frail and non-frail participants. The median survival time in frail and non-frail patients was 16.9 months and 29.9 months, respectively (log-rank test *p* = 0.024). Frailty was a significant predictor of all-cause mortality in the univariable analysis (hazard ratio (HR) = 1.69, 95%CI = 1.06–2.69, *p* = 0.028) and multivariable analysis (adjusted HR (aHR) = 1.68, 95%CI = 1.03–2.72, *p* = 0.037), along with palliative treatment intent (aHR = 2.53, 95%CI = 1.35–4.74, *p* = 0.004) (see multivariable model #1 in Table 2). The classification performance of the FI in multivariable analysis was moderate according to Uno’s c-statistic of 0.66 (Table 2).

Figure 1B shows the probability of all-cause mortality among patients with and without myosteatosis. The median survival time in patients with and without myosteatosis was 18.7 and 36.7 months, respectively (log-rank test *p* = 0.030). Patients with myosteatosis were approximately twice as likely to die of any cause (aHR = 2.14, 95%CI = 1.07–4.30, *p* = 0.032), along with patients undergoing palliative chemotherapy (aHR = 2.84, 95%CI = 1.49–5.40, *p* = 0.001) (see multivariable model #2 in Table 2). However, the classification accuracy of this model was also moderate based on Uno’s c-statistic of 0.66 (Table 2).

Multivariable model #3 included both the FI and myosteatosis as predictors of all-cause mortality (Table 2). The performance of the FI for predicting all-cause mortality was improved (aHR = 1.81, 95%CI = 1.11–2.94, *p* = 0.018) after inclusion of myosteatosis (aHR = 2.33, 95%CI = 1.16–4.69, *p* = 0.018) in this multivariable model (see multivariable mode #3 in Table 2). In addition to the FI and myosteatosis, a palliative treatment intent was also predictive of all-cause mortality (aHR = 2.85, 95%CI = 1.49–5.45, *p* = 0.002). The multivariable model with both the FI and myosteatosis displayed the highest predictive ability for all-cause mortality according to Uno’s c-statistic: 0.70 (Table 2). No interaction was found between the FI and myosteatosis.

## 4. Discussion

The objective of this retrospective analysis was to examine the ability of frailty and myosteatosis to separately predict all-cause mortality, as well as to explore whether myosteatosis adds value to frailty for predicting all-cause mortality in older patients initiating chemotherapy.

Most participants in our cohort (80%) had myosteatosis and 51.3% were frail. Among frail participants, 81.4% had myosteatosis. Previous work in older adults with cancer found a significant relationship between myosteatosis based on CT-derived SMD and the prevalence of frailty [21]. The relationship between frailty and myosteatosis might be explained, in part, by the findings of the Health ABC Study that showed a significant positive relationship between SMD and muscle strength in older adults [14]. Additionally, a higher degree of myosteatosis based on low SMD was found to be significantly associated with low walking speed in older frail individuals, and these associations were independent of levels of proinflammatory cytokines (c-reactive protein, tumour necrosis factor-α, and interleukin 6) [13]. Further work in patients with colorectal cancer demonstrated that low gait speed was a determinant of myosteatosis, and the severity of myosteatosis based on fat infiltration in the skeletal muscle was significantly associated with a higher prevalence of frailty [12]. From a mechanistic perspective, myosteatosis has a negative effect on muscle strength and walking speed via alterations in the orientation of muscle fibres [31], exacerbation of muscle protein breakdown [32], impairments in muscle contraction [33], and inflammation that further decreases the production of force [34]. Muscle strength and walking speed are among the principal characteristics of physical frailty [35]. Additionally, low walking speed is a measure of physical performance, a multidimensional concept that involves several systems (e.g., cardiovascular, respiratory, nervous, musculoskeletal) [13,24]. Therefore, low walking speed may be suggestive of impairments in several physiological systems, in line with the principles of frailty based on an FI [6].

We also found that myosteatosis was a consistent predictor of all-cause mortality in this mixed cohort of patients in terms of disease site, stage, and chemotherapy used. Specifically, myosteatosis at the L3 level was associated with a ~two-fold risk of all-cause mortality. Meta-analytic evidence in oncology corroborates our findings suggesting that fat infiltration in the skeletal muscle deserves clinical attention and downstream management strategies [10,36,37]. Myosteatosis appears to worsen insulin resistance and systemic inflammation [38,39,40] that may favour tumour growth and progression [41], in addition to the development of new comorbidities or the worsening of existing pathological syndromes [42], thereby increasing the risk of all-cause mortality. In addition to overall mortality, myosteatosis may be a predictor of adverse postoperative outcomes in geriatric oncology. Evidence from a two-centre study of 921 older adults awaiting colorectal cancer surgery demonstrated a higher risk of postoperative complications and length of hospital stay in the presence of myosteatosis [43]. These findings strengthen the rationale for further examining the value of myosteatosis in predicting clinical outcomes in older patients. The FI was also a predictor of all-cause mortality in this group of patients, in line with previous evidence in oncology [1]. Notably, the FI and myosteatosis each exhibited a comparable performance in predicting all-cause mortality according to Uno’s c-statistic. However, the addition of myosteatosis appears to improve the prognostic value of the FI (c-statistic from 0.66 to 0.70). The contribution of both frailty and myosteatosis to all-cause mortality in older adults with cancer may be mediated by the exacerbation of several pathways that include but are not limited to metabolic dysregulation [44,45], insulin resistance [16,17], oxidative stress [46,47], systemic inflammation [11,15], mitochondrial dysfunction [48,49] and increased muscle catabolism [32,48,49,50], all of which impair muscle morphology and function, leading to worse fatigue, inactivity, and a higher risk of all cause-mortality [51]. Further mediation analyses are required to investigate these potential mechanisms.

Currently, the assessment of frailty does not take into account the presence of myosteatosis. However, our findings suggest that myosteatosis may improve the prognostic ability of frailty (HR for FI in multivariable models increased from 1.68 to 1.81). An important question is whether and how clinicians can assess myosteatosis in daily clinical practice. It is important to acknowledge that quantification of myosteatosis through CT scans manually might not be feasible in daily clinical practice, predominately due to the time constraints. Additionally, depending on the anatomical location examined for determining the presence of cancer, not all patients have an abdominal CT scan (e.g., leukemia, lung cancer, head and neck cancer). Whether myosteatosis in other anatomical regions is associated with worse outcomes in older adults with cancer warrants further research. This is an important consideration as most studies have examined myosteatosis at the L3 level [10]. AI-based software tools may minimize the existing barriers to assessing myosteatosis in daily clinical practice [52]. Previous work has demonstrated that fully automated AI-based software (Visage Imaging GmbH, Version 7.1, Berlin, Germany) integrated into picture archiving and communications systems (PACS) can accurately assess body composition [52] and predict clinically relevant outcomes [53], yielding myosteatosis information readily available at the time of radiological report reviews.

Feasible and effective non-invasive strategies for preventing or reversing frailty and myosteatosis are important and may potentially improve clinically relevant outcomes. According to a network meta-analysis in individuals ≥ 60 years old, resistance training had the highest probability to be the most effective type of exercise for reducing frailty (surface under the cumulative ranking curve = 90.0%) [54]. Resistance training has also been shown to reduce myosteatosis based on muscle attenuation in older adults [37,55]. These findings highlight the role of exercise professionals (e.g., physiotherapist, exercise physiologist, kinesiologist) in prescribing personalized resistance training programmes to prevent or reverse frailty and/or myosteatosis in older adults with cancer.

Our findings should be interpreted cautiously due to the following limitations. First, given the retrospective nature of the study, we cannot infer causation between either myosteatosis or frailty and mortality. Second, the heterogeneity in cancer site, stage, and chemotherapy used, in addition to the modest sample size, might have affected our ability to accurately reflect the true relationships between all-cause mortality and the FI and myosteatosis. Another important limitation is that we did not capture physical activity status at baseline and during treatment or downstream strategies following chemotherapy that could potentially influence the risk of overall mortality. Additionally, the lack of proinflammatory markers from our analysis is an important limitation given that inflammation is associated with both frailty and myosteatosis, as well as all-cause mortality. Proinflammatory cytokines were not routinely assessed for older adults in the OACC and patients with mCRPC. Despite these limitations that impact the generalizability of our results, this study examined a novel and clinically relevant research question that warrants confirmation with larger studies.

## 5. Conclusions

CT-based myosteatosis at the L3 level improves the performance of frailty for predicting all-cause mortality in older adults with cancer. Efforts to prevent or reverse frailty and myosteatosis with exercise should be made after a cancer diagnosis. Our findings warrant further research in larger scale studies.

## Figures and Tables

**Table 1:** Characteristics of study participants at baseline.

Variable	Frail (n = 59)	Non-Frail (n = 56)	*p*	Missing Data (%)
Age (years), mean (SD)	78.1 (7.0)	76.1 (5.9)	0.10	0 (0)
Sex (males), n (%)	43 (72.9)	39 (69.6)	0.70	0 (0)
Treatment intent (palliative), n (%)	41 (69.5)	37 (66.1)	0.69	0 (0)
Disease site, n (%)			0.41	0 (0)
Genitourinary	33 (55.9)	29 (51.8)		
Gastrointestinal	6 (10.2)	11 (19.6)		
Gynecological	7 (11.9)	8 (14.3)		
Lymphoma	13 (22.0)	8 (14.3)		
Disease stage, n (%)			0.27	0 (0)
Localized	4 (6.8)	7 (12.5)		
Locally advanced	3 (5.1)	7 (12.5)		
Metastatic	39 (66.1)	34 (60.7)		
Hematologic	13 (22.0)	8 (14.3)		
Chemotherapy agent(s)			0.41	0 (0)
Alkylating	7 (11.9)	7 (12.5)		
Alkylating and monoclonal antibody	10 (16.9)	7 (12.5)		
Alkylating and taxanes	4 (6.8)	4 (7.1)		
Antimetabolites	5 (8.5)	2 (3.6)		
Antimetabolites and alkylating	4 (6.8)	9 (16.1)		
Antimetabolite and monoclonal antibody	0 (0)	1 (1.8)		
Antimetabolite and taxanes	0 (0)	2 (3.6)		
Taxanes	29 (49.2)	24 (42.9)		
Body mass index, mean (SD)	27.8 (4.5)	25.6 (5.4)	0.021	0 (0)
Comorbidities				0 (0)
Anxiety, n (%)	6 (10.2)	2 (3.6)	0.16	
Arrythmias, n (%)	10 (16.9)	1 (1.8)	0.006	
Arthritis, n (%)	29 (49.2)	13 (23.2)	0.004	
COPD, n (%)	6 (10.2)	5 (8.9)	0.82	
Coronary artery disease, n (%)	12 (20.3)	4 (7.1)	0.041	
Congestive heart failure, n (%)	4 (6.8)	0 (0)	0.047	
Depression, n (%)	5 (8.5)	1 (1.8)	0.10	
Diabetes, n (%)	22 (37.3)	5 (8.9)	<0.001	
Hearing impairment, n (%)	11 (18.6)	4 (7.1)	0.067	
Hyperlipidemia, n (%)	25 (42.4)	21 (37.5)	0.59	
Hypertension, n (%)	42 (71.2)	24 (42.9)	0.002	
Visual impairment, n (%)	9 (15.3)	2 (3.6)	0.033	
Osteoporosis, n (%)	13 (22.0)	7 (12.5)	0.18	
Valvular disease, n (%)	3 (5.1)	1 (1.8)	0.33	
Dependent in one or more IADLs, n (%)	41 (69.5)	20 (35.7)	<0.001	0 (0)
VES-13 (≥3), n (%)	42 (71.2)	23 (41.1)	0.001	0 (0)
Cognitive impairment ^a^, n (%)	30 (51.7)	18 (32.1)	0.034	1 (0.9)
Albumin (g/L), mean (SD)	37.2 (3.2)	39.7 (2.8)	<0.001	23 (20.0)
Alkaline phosphatase (u/L), Median (IQR)	101.5 (76.0–165.75)	82.5 (66.0–107.5)	0.11	1 (0.9)
Hemoglobin (g/L), mean (SD)	109.7 (19.9)	123.1 (15.2)	<0.001	1 (0.9)
Lactate dehydrogenase (u/L), mean (SD)	312.1 (121.8)	233.3 (62.8)	<0.001	1 (0.9)
Neutrophil-to-lymphocyte ratio (>3), n (%)	5.8 (4.7)	3.3 (1.9)	<0.001	1 (0.9)
Low grip strength per EWGSOP2, n (%)	32 (54.2)	8 (14.3)	<0.001	0 (0)
Low physical performance ^b^ n (%)	33 (55.9)	13 (23.2)	<0.001	0 (0)
Skeletal muscle density (HU), mean (SD)	26.4 (8.8)	29.2 (10.9)	0.066	0 (0)
Myosteatosis (present), n (%)	48 (81.4)	44 (78.6)	0.71	0 (0)

COPD = chronic obstructive pulmonary disease; EWGSOP2 = European Working Group on Sarcopenia in Older People 2; IADLs = instrumental activities of daily living; VES-13 = Vulnerable Elders Survey-13. ^a^ The Mini-Cog and Montreal Cognitive Assessment (MoCA) were used to determine cognitive impairment in older adults who underwent a geriatric assessment and older patients with mCRPC, respectively. The Mini-Cog was administered by a clinical nurse specialist (cutoff used: <4/5), whereas the MoCA was administered by a research coordinator (cutoff used: <25/30). ^b^ Low physical performance for older adults who underwent a geriatric assessment was defined as an SPPB score of ≤8/12. Low physical performance for older patients with mCRPC was defined as <0.8 m/s on the 4 m gait speed.

**Table 2:** Univariable and multivariable Cox regression on the impact of the FI and myosteatosis on all-cause mortality (n = 115).

Variable	Univariable HR (95%CI)	*p*	MV#1 ^c^ aHR (95%CI)	*p*	MV#2 ^d^ aHR (95%CI)	*p*	MV#3 ^e^ aHR (95%CI)	*p*
Age per 10-year increase	1.18 (0.83–1.67)	0.35	1.35 (0.96–1.90)	0.085	1.35 (0.95–1.91)	0.095	1.23 (0.87–1.73)	0.23
Sex (males)	1.53 (0.86–2.71)	0.14	1.26 (0.69–2.30)	0.46	1.08 (0.58–2.03)	0.81	1.06 (0.57–1.99)	0.85
Body mass index kg/m^2^			0.97 (0.92–1.02)	0.21	0.98 (0.93–1.02)	0.30	0.97 (0.92–1.01)	0.16
Treatment intent (palliative)	2.28 (1.27–4.10)	0.006	2.53 (1.35–4.74)	0.004	2.84 (1.49–5.40)	0.001	2.85 (1.49–5.45)	0.002
Frail (>0.25)	1.69 (1.06–2.69) ^a^	0.028	1.68 (1.03–2.72)	0.037	Not included		1.81 (1.11–2.94)	0.018
Non-frail (≤0.25)	ref.		ref.		ref.		ref.	
Myosteatosis (present)	2.04 (1.05–3.99) ^b^	0.036	Not included		2.14 (1.07–4.30)	0.032	2.33 (1.16–4.69)	0.018
Myosteatosis (absent)	ref.		ref.		ref.		ref.	

aHR = adjusted hazard ratio; MV = multivariable model, ^a^ Uno’s c-statistic: 0.58, ^b^ Uno’s c-statistic: 0.56, ^c^ Uno’s c-statistic: 0.66, ^d^ Uno’s c-statistic: 0.66, ^e^ Uno’s c-statistic: 0.70.

## Data Availability

Data are available from the corresponding author (SMHA), upon reasonable request. Email: shabbir.alibhai@uhn.ca.

## Supplementary Materials

The following supporting information can be downloaded at: https://www.mdpi.com/article/10.3390/curroncol31120578/s1, Table S1: Variables and thresholds used to create the FI.
