# Supplementary material for: The Role of Frailty and Myosteatosis in Predicting All-Cause Mortality in Older Adults with Cancer
Source: Curr Oncol. 2024 Dec 6;31(12):7852–62. doi: 10.3390/curroncol31120578 (PMC11674696; doi:10.3390/curroncol31120578)
Supplement: Supplementary file 1 [file curroncol-31-00578-s001.zip › curroncol-3203504-supplementary.pdf]

Supplementary Table

Supplemental Table S1. Variables and thresholds used to create the FI.

**Comorbidities (n=14)**	**Cut point**
Coronary artery disease including myocardial infarction	Yes= 1<br>No= 0
Congestive heart failure	
Arrhythmias	
Valvular disease	
Hyperlipidemia	
Visual impairment	
Hearing impairment	
Arthritis	
Osteoporosis	
Anxiety	
Depression	
Chronic obstructive pulmonary disease	
Diabetes	
Hypertension	
**BMI**	<18.5 or ≥30= 1<br>≥25 to 29.9= 0.5<br>18.5 to 24.9= 0
**Dependent in one or more IADLs**	Yes=1<br>No=0
**Cognitive impairment**	Mini-cog (OACC)<br>• Abnormal= 1<br>• Borderline/requires further testing= 0.5<br>• Normal= 1<br><br>MoCA (patients with mCRPC)<br>• 10-17= 1<br>• 18-25= 0.5<br>• ≥26= 0
**Grip strength (EWGSOP2)**	Males<br>• <27kg= 1<br>• ≥27kg= 0<br>Females<br>• <16kg= 1<br>• ≥16kg= 0
**Physical performance**	SPPB (OACC)<br>• ≤8= 1<br>• >8= 0<br>Gait speed (patients with mCRPC)

	• <0.8m/s=1<br>• ≥0.8m/s=0
**Blood markers (n=5)**	
Hemoglobin (g/L)	• <110=1<br>• ≥110=0
Neutrophil-to-lymphocyte ratio	• >3=1<br>• ≤3=0
Lactate dehydrogenase (u/L)	• >280=1<br>• ≤280=0
Albumin (g/L)	• <35=1<br>• ≥35=0
Alkaline phosphatase (u/L)	• >140=1<br>• ≤140=0

BMI = body mass index; EWGSOP2= European Working Group on Sarcopenia in Older People 2; IADLs= instrumental activities of daily living; mCRPC= metastatic castrate-resistant prostate cancer; OACC= Older adults with cancer clinic; SPPB= Short Physical Performance Battery
